# Analysis of the biofilm proteome of *Xylella fastidiosa*

**DOI:** 10.1186/1477-5956-9-58

**Published:** 2011-09-22

**Authors:** Mariana S Silva, Alessandra A De Souza, Marco A Takita, Carlos A Labate, Marcos A Machado

**Affiliations:** 1 Universidade Estadual de Campinas (UNICAMP), Campinas, SP, Brazil; 2 Centro APTA Citros 'Sylvio Moreira'(CCSM), Cordeirópolis, SP, Brazil; 3 Laboratório Max Feffer de Genética de Plantas, Departamento de Genética, Escola Superior de Agricultura 'Luiz de Queiroz', Universidade de São Paulo (USP), Piracicaba, SP, Brazil

## Abstract

**Background:**

*Xylella fastidiosa *is limited to the xylem of the plant host and the foregut of insect vectors (sharpshooters). The mechanism of pathogenicity of this bacterium differs from other plant pathogens, since it does not present typical genes that confer specific interactions between plant and pathogens (avr and/or hrp). The bacterium is injected directly into the xylem vessels where it adheres and colonizes. The whole process leads to the formation of biofilms, which are considered the main mechanism of pathogenicity. Cells in biofilms are metabolically and phenotypically different from their planktonic condition. The mature biofilm stage (phase of higher cell density) presents high virulence and resistance to toxic substances such as antibiotics and detergents. Here we performed proteomic analysis of proteins expressed exclusively in the mature biofilm of *X. fastidiosa *strain 9a5c, in comparison to planktonic growth condition.

**Results:**

We found a total of 456 proteins expressed in the biofilm condition, which correspond to approximately 10% of total protein in the genome. The biofilm showed 37% (or 144 proteins) different protein than we found in the planktonic growth condition. The large difference in protein pattern in the biofilm condition may be responsible for the physiological changes of the cells in the biofilm of *X. fastidiosa*. Mass spectrometry was used to identify these proteins, while real-time quantitative polymerase chain reaction monitored expression of genes encoding them. Most of proteins expressed in the mature biofilm growth were associated with metabolism, adhesion, pathogenicity and stress conditions. Even though the biofilm cells in this work were not submitted to any stress condition, some stress related proteins were expressed only in the biofilm condition, suggesting that the biofilm cells would constitutively express proteins in different adverse environments.

**Conclusions:**

We observed overexpression of proteins related to quorum sensing, proving the existence of communication between cells, and thus the development of structuring the biofilm (mature biofilm) leading to obstruction of vessels and development of disease. This paper reports a first proteomic analysis of mature biofilm of *X. fastidiosa*, opening new perspectives for understanding the biochemistry of mature biofilm growth in a plant pathogen.

## Background

*Xylella fastidiosa *is a slow growing Gram-negative bacterium involved in many economically important plant diseases, such as citrus variegated chlorosis (CVC) in sweet orange, Pierce's disease (PD) in grapevine and other species such as coffee and plum. In all cases, *X. fastidiosa *is transmitted by leafhoppers into the xylem vessel where it colonizes and blocks the movement of water and nutrients, causing typical disease symptoms according to the host.

It is generally accepted that microbial populations use cell attachment to adhere to solid supports, surfaces and particles where they grow and survive in the natural state [1]. Biofilms consist of intricate three-dimensional matrices containing channels, presumably to let nutrients diffuse in and waste products diffuse out [2]. During the biofilm development, a number of changes in gene regulation that cause the adhering cells to become phenotypically and metabolically distinct from their planktonic counterparts [3].

The mechanisms involved in the resistance of biofilm cells to antimicrobial agents are complex and only partially understood. Important factors include cell density, as well as the extent and duration of cell-to-cell contact, the concentrations of diffusible substances and/or the ability to establish concentration gradients of diffusible substances and oxygen availability.

In other Gram-negative bacteria, such as *Pseudomonas aeruginosa*, biofilm development and the expression of virulence factors are dependent on quorum sensing. Similarly, the requirement of a *X. fastidiosa *cell density threshold in the xylem for CVC development, as well as its occurrence as biofilms, suggests that synthesis of pathogenicity determinants by these bacteria is dependent on quorum sensing [4], a cell-cell communication mechanism which plays an important role in the virulence of many plant pathogenic bacteria [5].

*X. fastidiosa *must be able to adhere to both plant and insect hosts. To colonize the insect's foregut the bacteria needs to adhere to the insect tissue so that it can resist the high flow of the xylem sap passing through. In plants, adhesion to the xylem walls enables appropriate conditions for bacterial growth and biofilm formation. An important aspect of bacterial pathogenesis is cell aggregation (bacterium-bacterium interaction), which has been proposed to lead to vascular occlusion of the xylem, causing water and nutrient stress in the plant [6,7].

Most of the proteomic work performed on biofilm cells has consisted of comparing the crude protein patterns of sessile and planktonic organisms [8]. In *P. aeruginosa*, the transition from mature-stage biofilm to the dispersion stage resulted in a reduction in 35% of detectable proteins. According to their protein profiles, dispersed bacteria were closer to planktonic cells than to mature-stage biofilm organisms [9]. The most significant proteomic alterations were observed when planktonic bacteria were compared to mature biofilm cells or to dispersing biofilm cells, with more than 800 detectable proteins exhibiting more than a six-fold change.

The different proteomic investigations performed on biofilm bacteria have enabled the characterization of some up- and down-regulated proteins in sessile cells. These proteins can be distributed into three main classes. The first class involves membrane proteins that have been reported to have a substantial influence on attachment and may also play a role in early biofilm development [10]. It has been shown that the adhesion of *Escherichia coli *cells to hydrophobic surfaces activates the Cpx two-component signal transduction system involved in the modulation of curli fibers, bacterial structures intimately involved in adhesion and biofilm formation [11].

The second class includes proteins involved in metabolic processes, such as amino acid metabolism, carbon metabolism and cofactor biosynthesis, revealing that the central metabolism is affected by the sessile mode of growth. The third class includes proteins involved in adaptation and protection. While it is difficult to discriminate an expression tendency in proteins belonging to the first two classes, biofilm bacteria accumulate most adaptation proteins. It has been suggest that this general stress response initiated by growth within a biofilm might explain the resistance of sessile cells to environmental stresses.

In this work, we describe the phenotypic changes in the biofilm growth mode of a systemic plant pathogen, *X. fastidiosa*. Two-dimensional gel electrophoresis (2DE) was used to demonstrate phenotypic differences between biofilm cells and planktonic cells. Comparative analysis of the proteomes indicated that there were distinct differences between the protein profiles involving changes in proteins expressed for metabolism, motility, attachment and stress condition. The differential expressed genes were confirmed by real-time quantitative polymerase chain reaction.

## Materials and methods

### Bacterial strain and culture condition

The pathogenic strain 9a5c of *X. fastidiosa*, originally isolated from sweet orange CVC diseased trees (*Citrus sinensis *L. Osb.), was isolated and grown in PW medium [12]. The first colonies were observed between 10 and 15 days. To obtain cells in biofilm, primary colonies were transferred to a polypropylene tube containing 3 mL of PW broth. When the OD_600 nm _reached 0.3, the cells were transferred to a 1 L flask containing 300 ml of PW broth, previously described, to promote *X. fastidiosa *biofilm formation *in vitro *[13]. The sample was collected after 20 days, corresponding to the mature phase of *X. fastidiosa *biofilm [14], when the most abundant layer of biofilm formation was observed in the glass at the medium-air interface. The biofilm layer was scraped from the flask and washed by centrifugation at 8000 g for 5 min at 4°C with water and storage at -80°C for later processing.

To obtain *X. fastidiosa *in planktonic growth, cells not attached to the glass were transferred weekly to another flask until they completely lost their capacity to adhere to the glass surface. This characteristic is obtained after approximately 10 passages. The cells were collected after 10 days (stationary phase) and washed with water under the same conditions as the biofilm cells. Others studies with this bacteria related no changes in the mRNA levels suggesting that the regulation is slow in *X. fastidiosa*. For this reason we preferred to wait for the characteristic loss of the attachment ability to be sure that the expression of the proteins would be differential in biofilm *versus *planktonic cells, not for biofilm *per se*.

### Protein extraction

Proteins were extracted using acetone and trichloroacetic acid method. Total proteins from *X. fastidiosa *were extracted according to Damerval *et al*. [15]. The whole cell protein was homogenized with 10% trichloroacetic acid (TCA) in acetone. Proteins were precipitated for 1 h at -20°C. After centrifugation at 15000 g for 15 min, the protein pellets were rinsed with acetone containing 0.07% 2-mercaptoethanol for 1 h at -20°C. The supernatant was removed and protein pellet vacuum-dried and solubilized in 1 mL of solubilization buffer [7 M urea, 2 M thiourea, 0.4% (v/v) Triton-X 100, 4% (w/v) CHAPS, 50 mM DTE and 1% (v/v) Pharmalyte pH 3-10]. Proteins were quantified using the Bradford method [16].

### SDS PAGE

For the analysis of high molecular weight proteins, 8 μL (approximately 120 μg of protein) of whole cell protein extract was added to 8 μL of sample buffer 1-DE [6% w/v SDS (sodium dodecyl sulfate), 100 mM Tris (pH 6.8), 30% glycerol, 100 mM Dithiothreitol (DTT) and 0.001% w/v bromophenol blue (BPB)], boiled for 5 min and separated on 9%T polyacrylamide gels (14 cm × 16 cm × 0.15 cm) containing 10% glycerol. Proteins were visualized using Coomassie blue staining [17].

### Two-Dimensional Gel Electrophoresis

Protein samples (750 μg of protein/350 μL) were applied onto 3-10 non-linear immobilized pH gradient strips (18 cm, GE Amersham Biosciences). Strips were rehydrated for 12 h at room temperature at 50 V. Isoelectric focalizations (IEF) were performed on an IPGphor apparatus (GE Amersham Biosciences) at 75 KVh. After the IEF, the strips were kept at -80°C until needed. Before the second dimension, strips were kept at room temperature for 12 min in equilibration buffer [6 M urea, 2% (w/v) SDS, 50 mM Tris-HCl pH 6.8, 30% (v/v) glycerol, 0.001% (w/v) bromophenol blue] with 2% (w/v) DTT, and then, for 10 min with 4% (w/v) iodoacetamide (IAA). The second dimension was performed in vertical gradient 10-18% (w/v) polyacrylamide gels at 30 mA per gel until the dye reached the bottom of the gel. Three replicates were performed for each sample. Proteins were stained with Coomassie Brilliant Blue G-250 (CBB). Gels were fixed in a solution containing 40% (v/v) ethanol and 10% (v/v) acetic acid for 60 min and washed with water (2 × 10 min). For protein detection, the gels were kept overnight in staining solution [20% (v/v) methanol, 10% (w/v) ammonium sulfate, 2% (v/v) phosphoric acid and 0.1% (w/v) CBB]. After 3 washes in water (10 min each), the gels were stored in 1% (v/v) solution of acetic acid for image analysis and spot selection for sequencing [18].

### Image acquisition and analysis

The three replicates of the biofilm and planktonic 2-DE gels were scanned using a UTA-1100 scanner and Labscan v 6.0 software (GE Amersham Biosciences). Image analysis was performed automatically using Melanie software v.3 (GeneBio, Geneva, Switzerland). Image analysis steps included image filtration, spot detection and measurement, background subtraction, and spot matching. One biofilm gel and one planktonic gel served as the reference, and the spots of the other replicates were referenced to it. Initially, spots were automatically matched, and the positions of unmatched spots were then manually determined. The molecular mass (kDa) of each protein was estimated by comparison with those of a standard marker set, and the isoelectric points (pIs) were determined by the spot positions along the immobilized pH gradient strips [18].

### *In-gel *protein digestion

Protein spots were excised from the gels, cut into 1 mm cubes and washed with water for 15 min. For distaining, the gel pieces were washed several times with a solution of 50% (v/v) acetonitrile (ACN) and 50 mM ammonium bicarbonate, until complete removal of the CBB. The 2-DE gel spots were completely dehydrated with 100% (v/v) ACN, rehydrated with 20 mM DTT, and maintained for 40 min at 60°C. This solution was then discarded and replaced by 55 mM iodoacetamide before keeping the tubes, in darkness, for 30 min. The gel pieces were dehydrated again with 100% ACN and left to air-dry for complete removal of the solvent. The protein digestion was carried out with a solution of 10 ng μL-¹ trypsin (Promega) in 25 mM ammonium bicarbonate. The gel pieces were rehydrated with trypsin solution and the tubes were incubated for 12 h at 37°C. After digestion, the gel plugs were extracted twice with 50 μL of 60% (v/v) ACN, 1% (v/v) formic acid (FA) and once with 50 μL of ACN. All supernatants were combined and vacuum dried. Peptides were then suspended in 12 μL of 1% (v/v) FA for mass spectrometry analysis [18].

### Protein identification and mass spectrometry

Peptide mixtures were identified by on-line chromatography using a Cap-LC coupled to a Q-TOF Ultima API mass spectrometer (Waters). Five microliters of sample were loaded onto a nanoease-trapping column (0.18 mm × 23.5 mm, Waters) for pre-concentration, followed by peptide separation in a LC nanoease column Symmetry 300 C18 (3.5 μm, 75 mm × 100 mm, Waters). Peptides were eluted in a 60 min linear gradient of solvent B [95% (v/v) ACN, 0.1% (v/v) formic acid in water] at a flow rate of 250 nLmin-¹. Solvent A consisted of 5% (v/v) ACN and 0.1% (v/v) formic acid in water. All analysis was performed using a positive ion mode at a 3 kV needle voltage. The mass range was set from 300 to 2000 m/z, and the MS/MS spectra were acquired for the most intense peaks (≥ 15 counts). Multiply charged precursor ions were selected for fragmentation and peptide sequencing using automated data dependent acquisition (DDA) MassLynx software (Waters), switching from the MS to MS/MS mode and then returning to MS. The resulting fragmented spectra were processed using ProteinLynx v4.0 software (Waters) and the MASCOT MS/MS Ion Search http://www.matrixscience.com was used to compare the similarity of the sequences against the SwissProt and NCB/protein databases. Combined MS-MS/MS searches were conducted with a parent ion mass tolerance of 50 ppm, MS/MS mass tolerance of 0.2Da, carbamidomethylation of cysteine (fixed modification) and methionine oxidation (variable modification). According to MASCOT probability analysis, only significant (*P *< 0.05) hits were accepted [18].

### RNA isolation and cDNA synthesis

For the analysis of gene expression in biofilm and planktonic cells we followed the same procedure described for bacterial strain and culture condition. A fraction of the cells collected from the biofilm and planktonic growth was washed by centrifugation at 8.000xg for 5 min at 4°C with diethypyrocarbonate-treated water. The pellet was used for RNA extraction.

Total of RNA was isolated from *X. fastidiosa *cells using the RNeasy RNA extraction kit (Qiagen) and treated with the RNase-Free DNase Set (Qiagen). RNA concentration and its integrity were analyzed in the Agilent 2100 Bioanalyzer using a RNA Nano Labchips kit. For cDNA synthesis, a concentration of 300 ng of total RNA was mixed with random hexamers (Fermentas) in a final volume of 10 μl. Annealing was accomplished by incubation for 15 min at 75°C, followed by the addition of 5 μl SuperScript II reaction buffer (Fermentas), 1 μl of 0.1 M of DTT, 1 μl de dNTP mix (10 mM dATP, 10 mM dGTP, 10 mM dTTP, 10 mM dCTP), 1 μl of RNaseOut (40 U/μl) and 1 μl of SuperScript II reverse transcriptase (200 U/μl) to the reaction [19].

### Gene expression analysis by real-time RT-PCR

Total RNA was extracted from biofilm and planktonic cells grown in PW medium. Real-time RT-PCR was performed for each of the genes associated of the protein found in the 2-DE gel and for the endogenous control on cDNA templates prepared from the total RNA. The endogenous was chosen for having similar expression levels (P ≤ 0.05) in real time RT-PCR analysis of the *X. fastidiosa *growing in biofilm and planktonic cells, like the gene XF1740 and *pet*C. Reactions were prepared according to the following setup: 12.5 μl of Fast SYBR Green PCR master mix (Applied Biosystems), 50 ng of each primer, 1 μl of cDNA and water to 25 μl. The amplification condition was 1 cycle at 50°C for 2 min, 95°C for 10 min, followed by 40 cycles of 95°C for 1 s and 1 min at 60°C. The products of each primer set were also subjected to melt-curve analysis. The real-time RT-PCR was done using ABI PRISM 7500 Fast Sequence Detector System (Applied Biosystems). The results were analyzed with the ABI PRISM 7500 SDS using the relative quantification analysis (Applied Biosystems). Detection of the PCR products was measured by monitoring the increase in fluorescence caused by the binding of the SYBR green dye to double-stranded DNA. A fluorescence threshold was set automatically to 0.2. The endogenous control was used to normalize the samples for differences in the amounts of cDNA added to each reaction mixture. The results were normalized using the threshold cycle (Ct) obtained for the endogenous control present in the same plate. Ct is defined as the first amplification cycle at which fluorescence indicating PCR products is detectable above the threshold. For normalization, we utilized the equation: ΔCt = Ct (target gene)-Ct (endogenous control) and was done for two endogenous controls (XF1740 and *pet*C). The fold increase of the target gene in different growth of the bacteria was determined by the equation: Δ ΔCt = ΔCt (sample)-ΔCt (calibrator). The relative quantification was obtained by 2^- Δ ΔCt^. Statistical analysis were evaluated by ANOVA and compared by the t test (P ≤ 0.05) using the Assistat 7.3 beta software [19].

## Results

It has been reported that *X. fastidiosa *biofilm presents at least 5 phases of biofilm formation where 20 days corresponds to a mature biofilm [14]. That condition displays several characteristics known to confer advantages to the bacterial population. De Souza *et al *[14] observed an increase in the expression of genes involved in energy metabolism, regulatory functions, protein metabolism, plasmid maintenance and biosynthesis of amino acids, cofactors, surface polysaccharides, lipopolysaccharides, antigens and transport proteins in the same condition.

The proteins of mature biofilm and the planktonic cells of *X. fastidiosa *were compared after separation by one dimensional gel electrophoresis (SDS-PAGE) and Coomassie blue staining. The results obtained from the one-dimensional gel presented no relevant differences between biofilm and planktonic cells.

**Figure 1:**
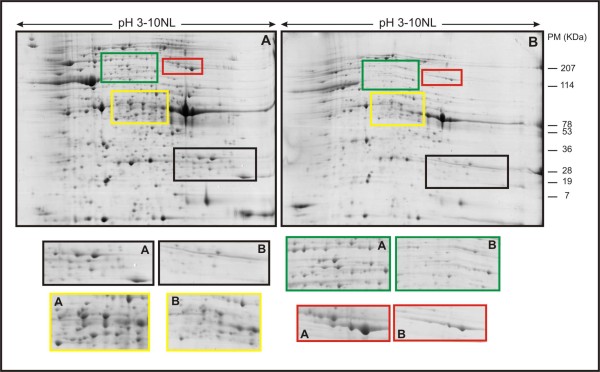
**2DE proteome patterns for mature biofilm cells (A) and planktonic cells (B) of *X. fastidiosa***. The horizontal axes represent pIs of the isoelectric focusing gradients, and the vertical axes represent molecular masses (MW). The squares show the difference in numbers of spots in the same MW, in compare the biofilm and planktonic cells.

For further characterization of differences in protein expression between the biofilm and the planktonic cells, high-resolution 2-DE of whole-cell protein extracts was performed (Figure 1). The reproducibility of separation of the total proteins was the same in all triplicate gels (data not shown). By matching and comparing the 2-DE proteomes, a total of 456 protein spots in the biofilm were observed in pH ranging from 3 to 10 after Coomassie blue staining. For the planktonic cells, 387 protein spots were observed. And of those, 144 protein spots were found differentially expressed in the biofilm condition.

We chose a total of 144 proteins spots were found differentially expressed in the biofilm condition for identification and functional analysis. A total of 41 spots were identified in the databank, which corresponded of 31 differentially expressed genes (1.41% of total genome of *X. fastidiosa*) were found to express protein in the biofilm. The genes are distributed throughout the different categories into functional groups according to the *Xylella *database http://aeg.lbi.ic.unicamp.br/xf, as summarized in Table 1. The N-terminal sequence of the 103 proteins spots did not display significant similarity to proteins in the database, probably because the proteins were not included in the database or because a protein spot contained more than one protein. Approximately 4% of the proteins did not show significant homologies and were classified as hypothetical proteins, using programs for protein localization site.

**Table 1:** Identified proteins from mature biofilm cell

Gene name	Function	Mass	pI	Score	GeneCateg
XF1259	PhosphoenolpyruvateSynthase [Xylella fastidiosa 9a5c]	87677	5.58	52	I.B.3

XF 1136	tryptophan repressor binding protein [Xylella fastidiosa 9a5c]	20453	6.29	104	I.C.2

XF 0253	electron transfer flavoprotein alpha subunit [Xylella fastidiosa 9a5c]	33021	5.55	52	I.C.3

XF2547	succinyl-CoA synthetase subunit beta [Xylella fastidiosa 9a5c]	41133	5.08	52	I.C.7

XF1143	F0F1 ATP synthase subunit beta [Xylella fastidiosa 9a5c]	50747	5.0	539	I.C.8

XF1143	F0F1 ATP synthase subunit beta [Xylella fastidiosa 9a5c]	50747	5.0	211	I.C.8

XF0389	two-component system, regulatory protein [Xylella fastidiosa 9a5c]	25364	5.27	404	I.D

XF1427	bifunctional N-succinyldiaminopimelate-aminotransferase/acetylornithine transaminase protein [Xylella fastidiosa 9a5c]	44116	5.47	104	II.A.1

XF0114	2,3,4,5-tetrahydropyridine-2-carboxylate N-succinyltransferase [Xylella fastidiosa 9a5c]	32566	6.0	136	II.A.2

XF1822	ketol-acid reductoisomerase [Xylella fastidiosa 9a5c]	40369	6.17	127	II.A.2

XF1821	acetolactate synthase 2 catalytic subunit [Xylella fastidiosa 9a5c]	62856	5.77	100	II.A.2

XF0762	deoxycytidine triphosphate deaminase [Xylella fastidiosa 9a5c]	21803	6.82	175	II.B.3

XF 1985	tRNA/rRNA methyltransferase [Xylella fastidiosa 9a5c]	26787	6.24	99	III.B.3

XF0428	tryptophanyl-tRNA synthetase [Xylella fastidiosa 9a5c]	48164	5.78	224	III.B.4

XF0239	polynucleotide phosphorylase/polyadenylase [Xylella fastidiosa 9a5c]	78268	5.56	282	III.B.6

XF0576	metallopeptidase [Xylella fastidiosa 9a5c]	78441	6.94	99	III.C.1

XF1605	peptidyl-prolyl cis-trans isomerase [Xylella fastidiosa 9a5c]	31590	5.66	63	III.C.1

XF 1605	peptidyl-prolyl cis-trans isomerase [Xylella fastidiosa 9a5c]	31590	5.66	80	III.C.1

XF2628	elongation factor Tu [Xylella fastidiosa 9a5c]	43077	5.48	389	III.C.1

XF2628	elongation factor Tu [Xylella fastidiosa 9a5c]	43077	5.48	76	III.C.1

XF2628	elongation factor Tu [Xylella fastidiosa 9a5c]	43077	5.48	354	III.C.1

XF2628	elongation factor Tu [Xylella fastidiosa 9a5c]	43077	5.48	363	III.C.1

XF1186	trigger factor [Xylella fastidiosa 9a5c]	48623	5.34	67	III.C.2

XF1186	trigger factor [Xylella fastidiosa 9a5c]	48623	5.34	143	III.C.2

XF0615	chaperonin GroEL [Xylella fastidiosa 9a5c]	57835	5.45	252	III.C.2

XF0615	chaperonin GroEL [Xylella fastidiosa 9a5c]	57835	5.45	61	III.C.2

XF0615	chaperonin GroEL [Xylella fastidiosa 9a5c]	57835	5.45	133	III.C.2

XF0138	leucyl aminopeptidase [Xylella fastidiosa 9a5c]	52147	6.07	77	III.C.3

XF0381	ATP-dependent Clp protease subunit [Xylella fastidiosa 9a5c]	95805	5.36	211	III.C.3

XF0256	glucose-1-phosphate thymidylyltransferase [Xylella fastidiosa 9a5c]	32757	5.70	164	IV.A.1

XF0343	outer membrane protein [Xylella fastidiosa 9a5c]	42433	8.45	379	IV.A.2

XF0343	outer membrane protein [Xylella fastidiosa 9a5c]	42433	8.45	99	IV.A.2

XF0343	outer membrane protein [Xylella fastidiosa 9a5c]	42433	8.45	266	IV.A.2

XF1633	twitching motility protein [Xylella fastidiosa 9a5c]	38644	6.43	50	IV.D

XF 1321	septum site-determining protein [Xylella fastidiosa 9a5c]	29033	5.10	360	V.B

XF 1137	NonF-related protein [Xylella fastidiosa 9a5c]	24763	5.55	165	VII.C

XF 2234	low molecular weight heat shock protein [Xylella fastidiosa 9a5c]	17848	5.55	75	VII.G

XF 0196	hypothetical protein XF0196 [Xylella fastidiosa 9a5c]	19940	6.92	139	VIII.A

XF2283	hypothetical protein XF2283 [Xylella fastidiosa 9a5c]	34449	5.91	193	VIII.B

XF0925	hypothetical protein XF0925 [Xylella fastidiosa 9a5c]	43291	6.21	67	VIII.B

XF1213	GTP-binding elongation factor protein [Xylella fastidiosa 9a5c]	71702	5.65	193	IX

## Discussion

Under the biofilm condition, we observed proteins involved in energy metabolism, biosynthesis of amino acids, attachment, pathogenicity and adaptation to environment. Sauer *et al*. [9] indicate that in *P. aeruginosa*, physiological changes in the transition from mature stage biofilm to planktonic growth resulted in a 35% reduction in the protein pattern. Protein based approaches suggest that a large number of genes are differentially regulated during biofilm development. On the contrary, transcriptome analysis led to the conclusion that only 1% (73 genes) of *P. aeruginosa *genes showed differential expression in biofilm and planktonic cells [20]. Whiteley *et al*. [20] assigned the identified genes into classes such as motility, attachment, translation and metabolism. The low number of genes altered in expression following bacterial adhesion was confirmed using DNA microarray technology where 79 genes (1.8% of the total genome) changed in the biofilm compared to planktonic growth [21]. In *X. fastidiosa*, the authors [14] compared the gene expression profile between biofilm and planktonic growth using DNA microarrays and found that gene expression in biofilm is different from that observed in planktonic cells. Many genes (approximately 9.18%) were up regulated in biofilm and these ORFs were distributed in several functional categories.

**Figure 2:**
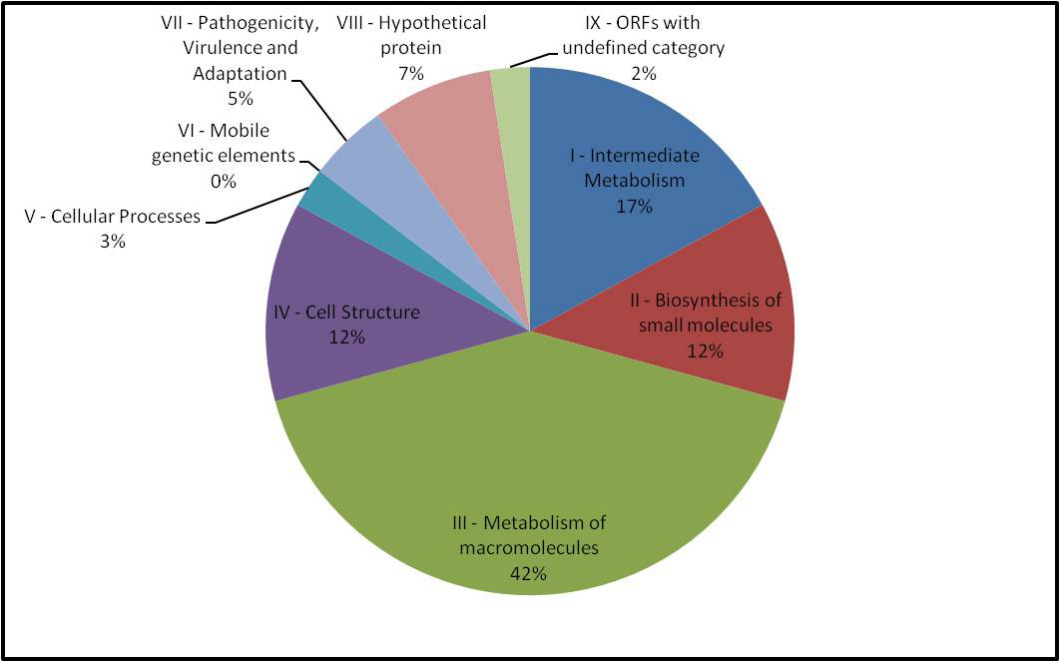
**Pie charts classifying the identified biofilm proteins according to biological function**. The identified proteins were grouped according to their biological processes and are expressed in percentage.

The proteomic investigation performed on biofilm bacteria enabled the characterization of some up-regulated proteins. These proteins can be distributed in different classes (Figure 2). One of them involves membrane proteins and has been reported by Coquet *et al*. [10] to have a substantial influence on attachment and also play a role in early biofilm development. In nonpiliated mutants of a *P. aeruginosa *strain, Vallet *et al*. [22] identified genes involved in bacterial adherence, like the genes specifying the components of a chaperone usher pathway involved in assembly of fimbrial subunits in microorganisms. This explains the overexpression of XF0615 when *X. fastidiosa *was in mature biofilm growth stage. We detected a two-component system regulatory protein (XF0389). In *E. coli *cells, the adhesion to hydrophobic surfaces activates the Cpx two-component signal transduction system that has been implicated in the modulation of the expression of curli, bacterial structures involved in adhesion and biofilm formation [11,23].

Some of the noteworthy changes in the outer membrane profiles point to the existence of environmental alterations within biofilms. The overexpression of *mop*B (XF0343) in *X. fastidiosa*, an anaerobically induced porin, suggests that biofilm cells are deprived of oxygen, as discussed by Sauer *et al*. [9].

Among the proteins involved in biofilm formation, the expression of PilC and PilA was not detected. Type IV fimbriae are known to be required for the initial attachment to the surfaces and these appendages act also in biofilm development [24,25], in this work we study a mature biofilm (the end of biofilm development). On the other hand, PilT, another protein of the type IV fimbriae is present in this condition of growth. This protein is responsible for retracting and extending the type IV fimbriae in a twitching motility and is critical in biofilm development. For *P. aeruginosa*, this cell structure is necessary for shaping the biofilm and for the development to a mature state [24]. This motility is important for colonization, allowing the bacteria to move around the plant vascular system [26] and start a new colonization on the other location of the xylem. Proteomic analysis of *P. putida *biofilm reveals an up regulation of type IV pili proteins, but down regulation of flagella proteins [9], those were important for a developed biofilm, not for a mature biofilm.

Another class of proteins up regulated in biofilm condition includes proteins implicated in the metabolic process, such as amino acid metabolisms, carbon metabolism and cofactor biosynthesis. Microorganisms have developed a mechanism to sense the bacterial population so that they can react to their changing environment, a phenomenon called quorum-sensing (QS). Gram-negative bacteria usually produce acylated homoserine lactones (AHLs) as QS signals. These molecules accumulate in function of the cell density and, above certain threshold, they trigger the expression of QS regulated genes. QS is an important mechanism for biofilm formation as this cell-cell communication system enables biofilms to respond as an organized group of bacteria [27], and plays an important role in the virulence of many plant pathogenic bacteria. In the present work, the overexpressed genes XF1605 and XF1186, peptidylprolyl *cis-trans *isomerases, were involved in the folding and degradation of proteins [28] and involved in QS, which is involved in the production of DSF (cis-11-methyl-2-dodecenoic acid) in many species of *Xanthomonas *and has been linked to the regulation of virulence, motility, toxin production, aerobic respiration, biofilm dispersal, extracellular enzyme and extracellular polysaccharide (EPS) production [5]. DSF was required for biosynthesis of a peptidyl-prolyl *cis-trans *isomerase (PPIase) that accelerated the rate limiting isomerization of *cis-trans *peptidyl-prolyl bonds during protein folding and possessed a chaperone-like function [5]. Overexpression of proteins was observed to be associated to quorum sensing and pathogenicity of the bacterium. Because of the condition of mature biofilm, there is high cell-cell communication and structure of the biofilm, leading to vessel blockage and disease development, and it is possible to the activated QS mechanism in biofilm.

**Figure 3:**
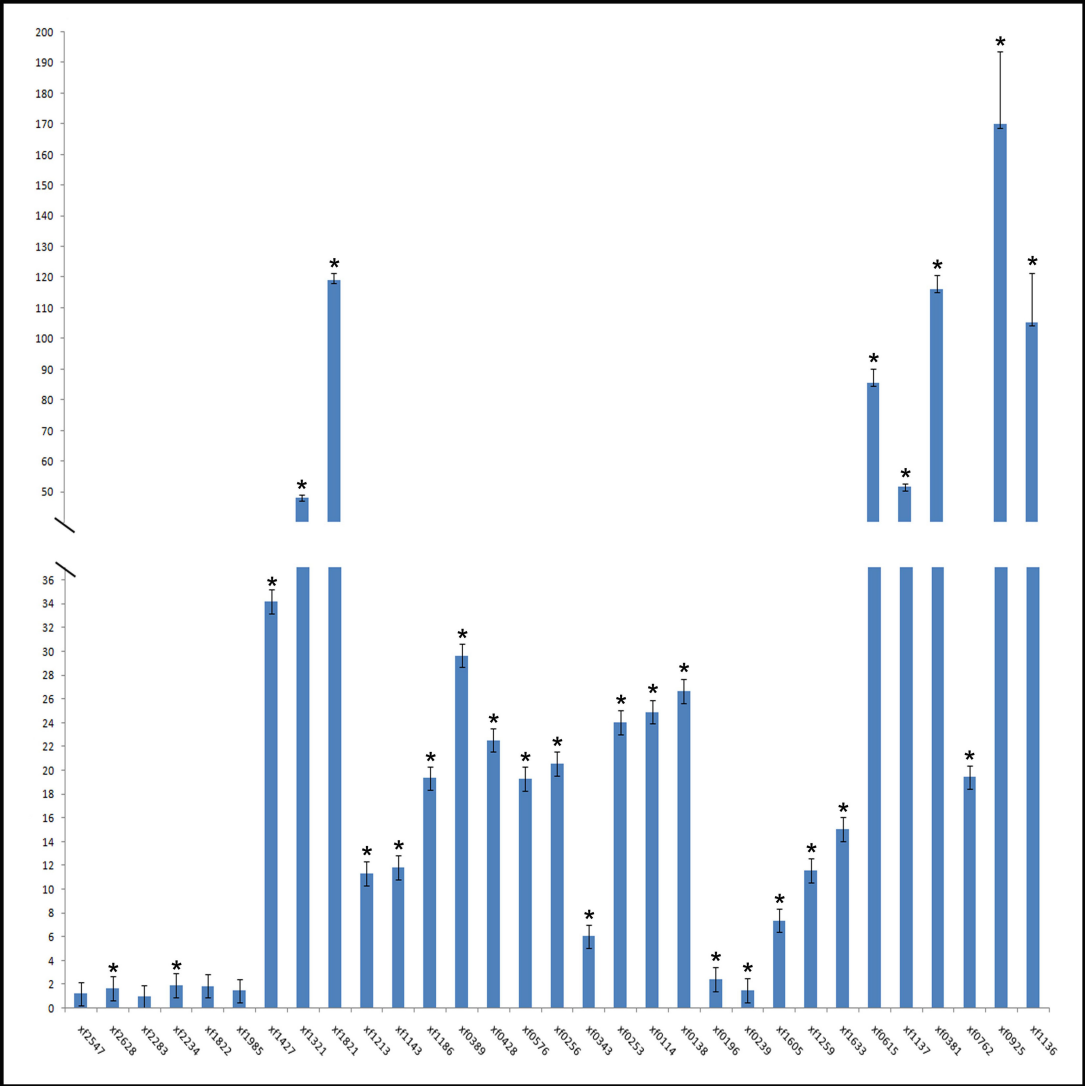
**Relative quantitation of genes by real-time quantitative polymerase chain reaction**. The samples were used for quantitation in the ABI 7500 Sequence Detector System (Applied Biosystems). The measures were normalized using threshold cycles (Ct) obtained for the amplifications of the endogenous control run in the same plate. The values represent the fold increase in gene expression compared with the values obtained for cDNA from planktonic cells (calibrator). The results are averages of three biological repetitions. Asterisk indicates significant difference (P ≤ 0.05) between the mean values compared to the control.

In the 2DE protein profile it was possible to observe the presence of the cellular protein XF0615 (*gro*EL), of the 60 kDa chaperone family, which promotes refolding of misfolded polypeptides, especially under stressful conditions and high cell density. Many bacteria have multiple copies of the *gro*EL gene, which is active under different environmental conditions [18]. The other protein, XF2628, an elongation factor, has an essential function in the elongation phase of mRNA translation and promotes GTP-dependent binding of aminoacyl-tRNA, as does XF1213, to the A-site of ribosomes during protein biosynthesis, that explain the post translational modification in the genes expression. A combined transcriptome and proteome analysis of *E. coli *during the high cell density culture showed the some proteins were up regulated as observed in planktonic cells. Interestingly, the patterns of gene expression observed by proteome and transcriptome analysis were mostly similar [29], like was for *X. fastidiosa *(Figure 3), when only few genes were expressed similarly in biofilm and planktonic cells, and the majority was differentially expressed.

A polynucleotide phosphorylase (XF0239) degrades plant chloroplasts to provide carbon sources or to facilitate the migration of the bacteria within xylem vessels [30]. XF0138 belongs to a cytosol aminopeptidase family and plays a key role in protein degradation and in the metabolism of biologically active peptides. The protein XF0576 is a predicted metalloendopeptidase and the role is post-translational modification, protein turnover and chaperones. XF1186 promotes the folding of newly synthesized proteins and XF1321 participates in cell division and chromosome partitioning [26].

The third class of proteins in the biofilm condition includes those involved in adaptation and protection. Most adaptation proteins are accumulated by biofilm bacteria, strengthening the idea that these proteins are expressed in biofilm showing high cell density and could be conferring advantages to the bacterial population like an inherent resistance to environmental factors that could harm the biofilm. In this sense, we observed the expression of toxin productions (XF1137), which are frequently referred to as virulence factors in bacterial pathogens [26,31]. The other gene, XF2234, expresses a low molecular weight heat shock protein (sHsp). sHsps are small stress induced proteins generally active as large oligomers consisting of multiple subunits, and are believed to be ATP-independent chaperones that prevent aggregation and are important in refolding in combination with other Hsps. Koide *et al*. [30] observed the induction of the gene XF0615, which encodes proteins from different Hsp families that are involved in the heat shock response and activated during environmental stress conditions for organism adaptation. We also found XF1213, a predicted membrane GTPase, which is involved in stress response in a signal transduction mechanism. The other protein observed in this category is the product of the gene XF0389 (popP or feuP or phoP), which is required for virulence in several bacterial species, such as *Samonella *and the plant pathogen *Erwinia carotovora *[32].

## Conclusions

*Xylella fastidiosa *biofilm was grown in the glass at the medium-air interface and its proteome compared to that planktonic grown using 2-DE electrophoresis. Using this technique, a total of 41 spots were identified using mass spectrometry.

Genes related to fimbrial and nonfimbrial adhesins (*xad*A1, *xad*A2, *pil*A2, *pil*C) genes encoding hemagglutinin-like secreted proteins and genes involved in exopolysaccharides (EPS) production were not found differentially expressed only in the biofilm condition. These genes were probably expressed in both growth conditions or overexpressed during phases before 20 days of biofilm formation [25]. It was suggested that these proteins might play a role in mediating cell-cell aggregation to form colonies and contribute to the biofilm maturation process in *Xylella*.

Some of the proteins identified in this study confer advantage for cells living in biofilm. Changes in transcription levels alter significantly the levels of proteins in the cells and that could greatly affect the biological response.

The proteins expressed in the biofilm of *Xylella *do not differ from the proteins of other bacterial species like *P. aeruginosa *and *E. coli*, corroborating the idea that proteins associated with adaptation and competitiveness are important factors for the maintenance of biofilms.

## Competing interests

The authors declare that they have no competing interests.

## Authors' contributions

MSS carried out the proteomics experiments, bioinformatic analysis and was involved in drafting the manuscript. AAS, MAT, CAL and MAM made substantial contributions to the study conception and design and critically revised the manuscript for intellectual content. All authors edited the manuscript and approved the final version.
